# Electrochemically Etched Tapered-Tip Stainless-Steel Electrospray-Ionization Emitters for Capillary Electrophoresis–Mass Spectrometry

**DOI:** 10.1021/acs.jproteome.3c00076

**Published:** 2023-03-03

**Authors:** Jordan T. Aerts, Per E. Andrén, Erik T. Jansson

**Affiliations:** † Department of Pharmaceutical Biosciences, Uppsala University, SE-751 24 Uppsala, Sweden; ‡ Science for Life Laboratory, Spatial Mass Spectrometry, Uppsala University, SE-751 24 Uppsala, Sweden

**Keywords:** electrochemical etching, tapered tip, stainless-steel emitter, electrospray ionization, CE−MS

## Abstract

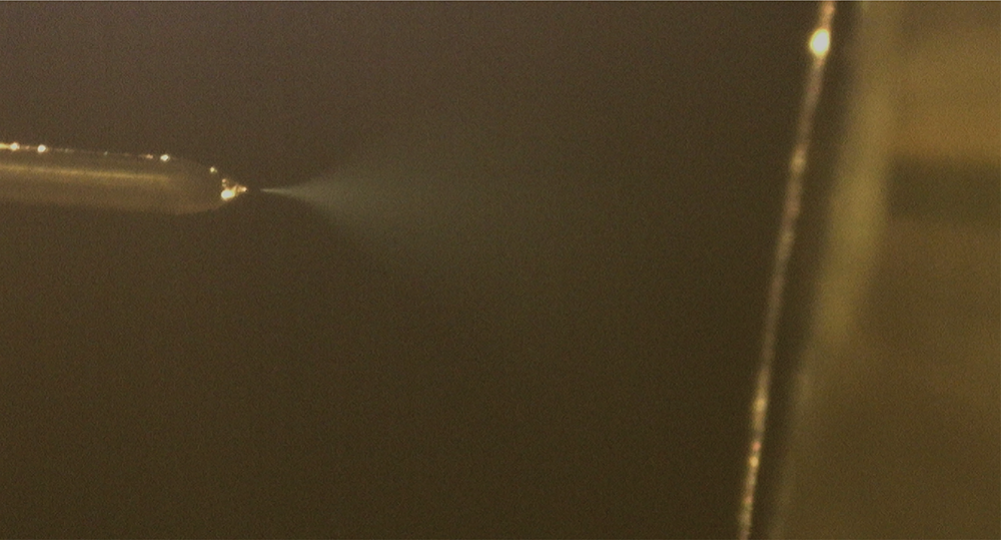

We have used household consumables to facilitate electrochemical etching of stainless-steel hypodermic tubing to produce tapered-tip emitters suitable for electrospray ionization for use in mass spectrometry. The process involves the use of 1% oxalic acid and a 5 W USB power adapter, commonly known as a phone charger. Further, our method avoids the otherwise commonly used strong acids that entail chemical hazards: concentrated HNO_3_ for etching stainless steel, or concentrated HF for etching fused silica. Hence, we here provide a convenient and self-inhibiting procedure with minimal chemical hazards to manufacture tapered-tip stainless-steel emitters. We show its performance in metabolomic analysis with CE–MS of a tissue homogenate where the metabolites acetylcarnitine, arginine, carnitine, creatine, homocarnosine, and valerylcarnitine were identified, all with basepeak separated electropherograms, within <6 min of separation. The mass spectrometry data are freely available through the MetaboLight public data repository via access number MTBLS7230.

## Introduction

Electrospray-ionization emitter tips used in mass spectrometry are usually made by blunt-end cutting either fused-silica or stainless-steel tubes; or by pulling a tubular resin under heat to produce a fine tip, such as borosilicate glass, or fused silica.^1−7^ In addition to these types of emitters, tapered-tip emitters have been deemed very useful for electrospray ionization for approaches to achieve interfacing of capillary electrophoresis to mass spectrometry.^8−12^ While pulled emitters are routinely made with borosilicate glass and fused-silica capillaries, this technique is not suitable for making stainless-steel emitters for electrospray ionization. For applications that require stainless-steel emitters, production of these are limited to mechanical cutting that also involve deburring and grinding down any roughness of the surface to achieve a straight and blunt tip. For production of tapered-tip stainless-steel emitters, even more mechanical craftsmanship is required to achieve the desired tip shape. This manual handling unfortunately often yields an asymmetrical taper of the stainless-steel emitter due to the mechanical properties of the material.

Previous work from the Smith group showed that it is feasible to make tapered-tip fused-silica capillaries by immersing the capillary into a beaker filled with HF, acting as an etchant of fused silica, while flowing H_2_O through the capillary.^8,10^ This is a very elegant method for two reasons: First, the flow of H_2_O from the capillary outlet, immersed in HF, prevents access of the etchant (HF) to the inner lumen of the capillary. Second, due to the surface tension properties of a liquid in contact with a solid object, HF creeps up on the outer wall of the fused-silica capillary, above the level of the meniscus of the liquid. In combination, these two features of this tip-producing method result in a self-inhibiting etching procedure where a tapered tip is obtained once all fused silica below the meniscus of HF in the beaker has been etched away.^8,10^

While tapered-tip fused-silica capillaries are very useful for a wide range of applications, the use of tapered-tip stainless-steel emitters have recently been shown to be of great benefit for sheath-flow assisted electrospray ionization for connection of capillary electrophoresis to mass spectrometry, providing up to 100-fold improved sensitivity when compared to blunt tip emitters.^11,12^ Here, we present how a tapered stainless-steel emitter may be produced with the use of materials and chemicals available as household products: oxalic acid and a USB power adapter. The electrochemical reaction used here for production of electrospray-ionization emitters is turned on, is left for ∼1 h and eventually the etching process self-inhibits, producing symmetrical tapered tips and removing the need for manual grinding and deburring of the emitter.

## Materials and Methods

### Chemicals

Oxalic acid was obtained from Merck (Darmstadt, Germany). Austenitic stainless-steel hypodermic needles (SS 304, 235 μm OD 108 μm ID, Hamilton, Reno, NV, or 270 μm OD 160 μm ID, G. Kinnvall AB, Sparreholm, Sweden) were connected via a FEP tubing sleeve (F-240 IDEX Health and Science, Oak Harbor, WA) and a 1/16 inch union to a syringe pump (Pump 11, Harvard Apparatus, Holliston, MA) that supplied water at 0.1 μL/min. A 5 W USB power adapter (Samsung, Seoul, South Korea) was used to drive the electrochemical etching.

### Etching Procedure

The anodic (*V*_+_) and cathodic (*V*_–_) leads of a USB cable were connected to the stainless-steel needle and a platinum wire, respectively. With the syringe pump already turned on for water supply through the stainless-steel needle outlet, the stainless-steel needle was immersed 2 mm into 1% oxalic acid (aq). Next, the platinum wire was in turn immersed into the oxalic acid solution, and the USB cable connected to a 5 W USB adapter supplying 5 V was plugged into a 220 V AC power strip with a residual current circuit breaker.

### Chemical and Electrical Safety

A solution of 1% oxalic acid has a pH of 1.3 and is harmful to skin and eyes. Personal protection equipment should include a laboratory coat, nitrile rubber gloves, and safety goggles. Electrical safety must be considered even when operating at low voltages. Do not touch any live wires. Avoid to connect the electrochemical cell onto the same electrical circuit as any instrumentation.

### Capillary Electrophoresis–Mass Spectrometry

Capillary electrophoresis separations were performed and gas phase ions generated using a coaxial sheath-flow CE-ESI interface previously described elsewhere.^13−15^ Metabolites were extracted from tissue samples prepared from a previously published biobank as previously described elsewhere.^16^ A brief description is given below.

#### Capillary Electrophoresis

Direct current high voltage power supply model HPS100-40-0.4 (Beijing Excellent Innovate HD Electronics Co., Ltd., Beijing, China) was operated at 15–26 kV in anode to cathode mode. Fused-silica capillaries of length 50–100 cm, 40 μm inner diameter, and 140 μm outer diameter (Trajan Scientific and Medical, Victoria, Australia) were used for analyte separation. Hydrodynamic sample injection volumes of the tissue-extracted metabolite solution ranged from 6–37 nL; the electrical circuit was connected to earth ground via the stainless-steel needle of the sheath-liquid syringe; the fused-silica capillary used for analyte separation was conditioned between injections with 3 column volumes H_2_O, 3 column volumes 0.1 M NaOH, 5 column volumes H_2_O, followed by 3 column volumes of background electrolyte [1% formic acid (aq)] delivered at 20 psi of house N_2_ (g) by connecting the separation capillary inlet to a in-laboratory built acrylic holder for solution vials using P-683 1/4–28 male to luer lock assemblies (Idex Health and Science, Oak Harbor, WA).

#### Electrospray Ionization

A 55 mm long 260 μm inner diameter, 360 μm outer diameter fused-silica capillary (Trajan Scientific and Medical, Victoria, Australia) was connected to a PEEK tee and attached to a microtight PEEK union assembly (P-727 and P-720, respectively, Idex Health and Science, Oak Harbor, WA) to allow the use of the NanoLockSpray source (Waters Corporation, United Kingdom) as a positioning stage at the mass spectrometer inlet. A tapered-tip stainless-steel emitter made according to the description above, made from stainless-steel hypodermic tubing (270 μm OD 160 μm ID, G. Kinnvall AB, Sparreholm, Sweden) was used for the electrospray-ionization emitter assembly. A voltage of 1.5–2.5 kV was applied and controlled by MassLynx software (Waters Corporation, Manchester, U.K.) directly to the tapered-tip stainless-steel emitter to generate ions. The sheath-liquid flow rate was 500 nL/min of 60% MeOH, 0.1% formic acid (aq).

#### Mass Spectrometry

Generated ions were analyzed on a Synapt G2-Si (Waters Corporation, Manchester, United Kingdom) operated in positive ion mode within a mass range of 50–1000 Da; the instrument was calibrated on the day of use using a 20 psi infusion of 0.01 M NaOH through the separation capillary to generate sodium formate clusters. The fragmentation spectra were generated in data dependent acquisition mode using a trap collision energy ramp from 5–50 eV. A quadrupole precursor ion selection window of ±10 mDa was used for mass spectrometry analysis and a previously determined analyte migration order^17^ was used for providing migration time values for the inclusion list for data dependent acquisition.

## Results

**Figure 1:**
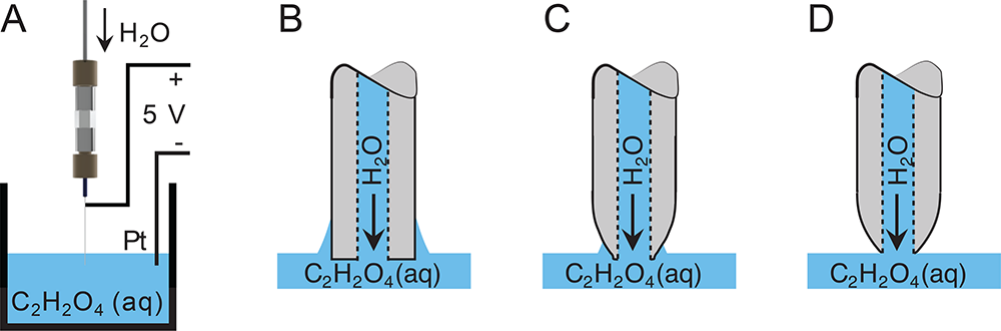
Schematic setup of the etching process shown in panel A with panels B–D highlighting in detail the interface of the needle-tip immersed into the etchant. (A) An SS 304 needle was connected through PEEK fittings and union to a syringe pump providing H_2_O. The needle was connected to the anodic lead of a USB power cable, and the needle-tip was immersed into a beaker containing 1% oxalic acid [C_2_H_2_O_4_ (aq)]. A platinum (Pt)-wire was attached to the cathodic lead of a USB power cable and immersed into the same beaker as the stainless-steel needle. Connecting the USB power adapter to a power strip with a residual current circuit breaker provides in turn 5 V to the electrochemical cell and initiates the etching of the stainless-steel needle. (B) When the needle-tip is immersed into oxalic acid, the surface tension of the liquid makes a small amount of liquid to climb up on the outer wall of the tip. Hence, some etching will occur above the level of the meniscus. (C) As the reaction proceeds, a larger amount of steel is etched away below the liquid surface compared to above. (D) Finally, the etching process self-inhibits once all stainless-steel material below the surface of solution of oxalic acid has been etched away and there is no longer any substantial thin film present above the liquid surface on the outer wall, rendering a tapered tip on the needle.

**Figure 2:**
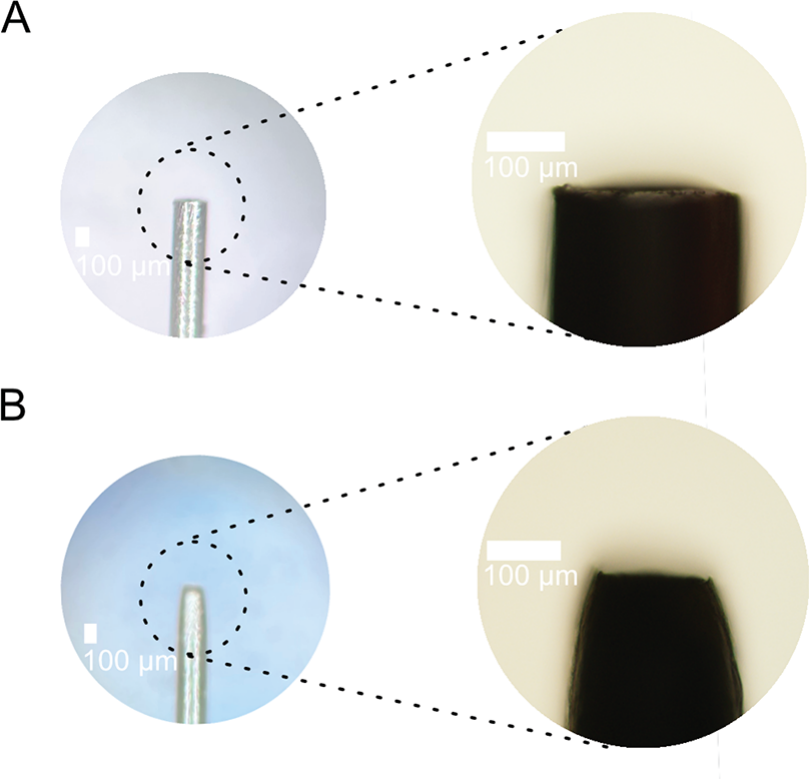
Stereomicroscopic images (and zoomed-in inserts obtained with a transmission light microscope) of (A) an unetched stainless-steel capillary and (B) an electrochemically etched stainless-steel capillary using 1% oxalic acid and a voltage of 5 V supplied by a commercial USB power adapter. Etching of OD 235 μm ID 108 μm austenitic stainless-steel hypodermic needles resulted in OD 140.0 ± 8.4 μm wide tapered-tip emitters with ∼16 μm wall thickness (*n* = 3).

Due to the anticorrosive nature of stainless steel, chemical etching can be difficult. In particular, austenitic stainless steels typically used for hypodermic tubing, and in turn, electrospray emitters, have high amounts of chrome and nickel, rendering them even harder to etch than other types of stainless steel. However, electrochemical reactions using oxalic acid and low-voltage power supplies have for a long time provided an approach to facilitate etching of austenitic stainless steel; the details of the etching process have previously been described elsewhere.^18^ Here, once the precut stainless-steel needle was immersed into the electrochemical cell filled with 1% oxalic acid, the reaction initiated rapidly with dramatic bubbling at the platinum wire (Figure 1A). The reaction eventually self-inhibited once the part of the stainless-steel needle immersed into the oxalic acid had been electrochemically etched away. This is due to the surface tension of the etchant, where at first the contact angle between the liquid meniscus and the initially straight, blunt-ended needle allows the etchant to creep up alongside the outer wall of the needle. Since less etchant is in contact with the outer wall above the meniscus of the etchant compared to below, the etching rate is faster below the meniscus (Figure 1B). As the etching proceeds, a tapered tip begins to form and the thin liquid film on the outer wall of the needle begins to recede down toward the meniscus (Figure 1C). Eventually, the tapering of the needle has gone so far that no liquid is able to creep up on the outer wall above the meniscus and all metal that initially was submerged into the etchant is gone (Figure 1D). At this point, the reaction stops. As such, the etching of stainless-steel needles is self-inhibitory, similar to what has been shown for fused-silica capillaries.^8,10^Figure 2 shows the shape of the unetched and electrochemically etched stainless-steel emitter. The terminus of the tapered-tip was 140.0 ± 8.4 μm wide, indicating a wall-thickness of ∼16 μm at the tip (the inner diameter of the emitter was 108 μm), and a taper angle of 13.3 ± 0.53° (*n* = 3).

**Figure 3:**
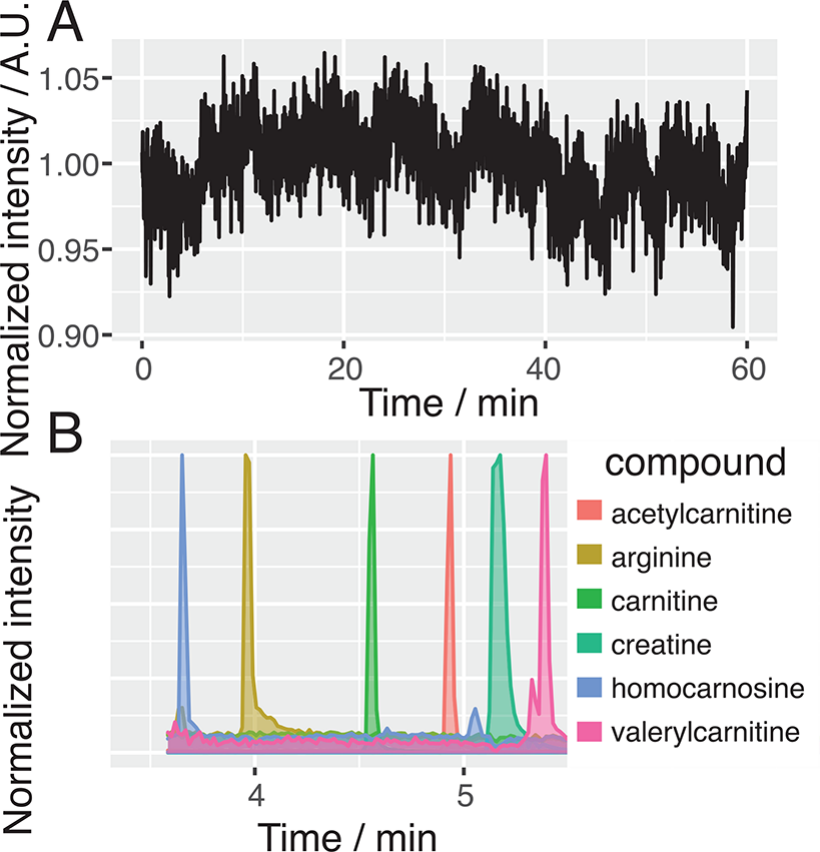
Tapered emitters were assessed for spray stability and used in CE–MS analysis of small molecules. (A) The emitter provided a spray with 2.3% RSD noise in the total ion electropherogram across 60 min when used as sheath-flow provider for CE–MS. The raw signal was normalized by its mean intensity across the depicted range to better visualize the spray stability. (B) The emitter was used for analysis of small molecules from tissue homogenate with symmetric peak shapes. The extracted ion electropherograms for the different compounds are defined as acetylcarnitine, *m*/*z* = 204.12; arginine, *m*/*z* = 175.12; carnitine, *m*/*z* = 162.11; creatine, *m*/*z* = 132.08; homocarnosine, *m*/*z* = 241.13; and valerylcarnitine, *m*/*z* = 246.17. The maximum intensity for each compound was normalized to unity for the purpose of clear visual representation of the electrophoretic migration times.

Further, we assessed the spray-stability of the tapered-tip emitters with mass spectrometry and used it for analysis of small molecules (metabolites) from tissue homogenates (Figure 3). We found the emitter to provide a stable spray across 60 min of use for our coaxial sheath-flow CE–MS interface, with only 2.3% RSD noise (Figure 3A). Use of the tapered-tip emitter resulted in symmetrical peak shapes in the extracted ion electropherograms (Figure 3B).

## Conclusions

We have provided a simple way to enable easy manufacturing of tapered-tip stainless-steel emitters for electrospray ionization. The method provides a safe way to produce emitters and remove the need for chemical hazards involving strong acids (such as concentrated HNO_3_ otherwise used for etching stainless steel). Further, the chemical etching requires fewer skills in craftsmanship such as grinding and deburring very small metal items. In addition, tapering and deburring emitters by hand may also yield asymmetrical tips. In contrast, our method provide symmetrical tips with a very convenient self-inhibiting electrochemical-etching reaction. Furthermore, we suggest the possibility our method provides to etch tapered-tip emitters completely off the power grid if desired. This could be done if a rubber-band tensely wrought around the piston of a syringe drives the delivery of H_2_O instead of a syringe pump, and a USB powerbank or other type of battery is used instead of a power adapter that plugs into a wall outlet. Since our method facilitates easy and fast fabrication of tapered-tip stainless-steel emitters, we consider the method to become useful for many research groups who already use or who would like to use this type of emitter for their own experimental work.

## Data Availability

The mass spectrometry data is freely available through the MetaboLight public data repository via access number MTBLS7230.
